# Hypertrophic Obstructive Cardiomyopathy Present as Acute Myocardial Infarction in a Nonagenarian

**DOI:** 10.1016/j.jaccas.2026.107190

**Published:** 2026-03-03

**Authors:** Xiaojing Wan, Yuji Zhan, Zeyu Gao, Ying Sui, Jun Ren, Haiwei Chen, Yan Chang, Chunyan Zhang, Yabin Wang

**Affiliations:** a Graduate School of the Chinese PLA General Hospital, Beijing, China; b 6th Department of Health Care, The Second Medical Center & National Clinical Research Center for Geriatric Diseases, Chinese PLA General Hospital, Beijing, China; c Department of Cardiology, The Sixth Medical Centre, Chinese PLA General Hospital, Beijing, China

**Keywords:** acute coronary syndrome, myocardial infarction, myocardial ischemia

## Abstract

**Background:**

Hypertrophic obstructive cardiomyopathy (HOCM) may appear as acute myocardial infarction in patients, posing diagnostic challenges.

**Case Summary:**

A 95-year-old male presented with dizziness, hypotension, and electrocardiogram changes suggesting left main disease, alongside a dynamic rise in cardiac biomarkers. Coronary computed tomography angiography (CCTA) and CT-derived fractional flow reserve (CT-FFR), revealed no functionally significant ischemia. Physical examination showed a systolic murmur, followed by echocardiography, which confirmed HOCM by demonstrating asymmetric septal hypertrophy, systolic anterior motion of the mitral valve, and a left ventricular outflow tract gradient of 40 mm Hg. Management with bisoprolol and mavacamten led to significant symptomatic improvement.

**Discussion:**

This case emphasizes that HOCM may present like acute myocardial infarction. Physical examination and multimodal cardiac imaging are crucial to avoid misdiagnosis.

**Take-Home Messages:**

HOCM is a differential diagnosis for acute coronary syndrome in elderly patients. Cardiac auscultation is crucial in differential diagnosis, combined with multimodal cardiac imaging.

## Graphical Abstract

**Figure:**
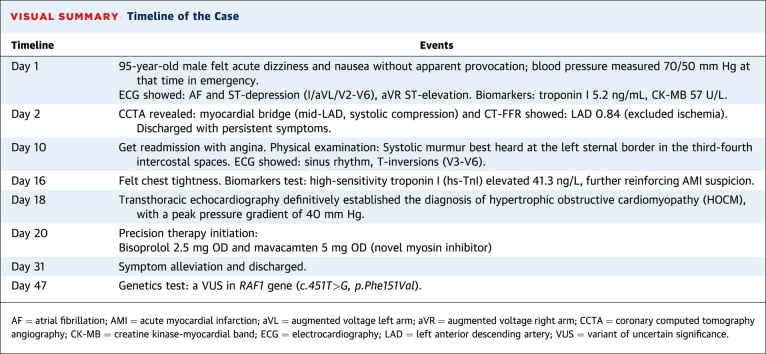

## History of Presentation

**Figure 1:**
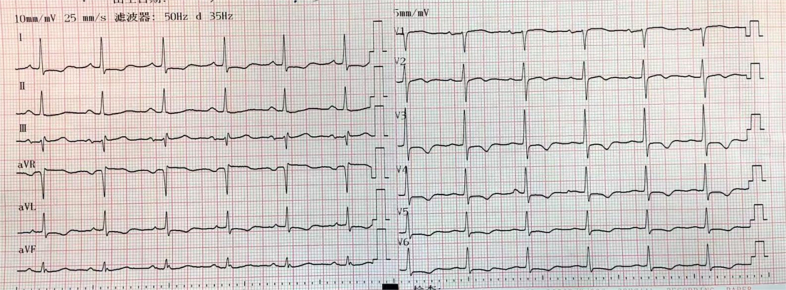
Electrocardiogram Obtained During Admission Demonstrating Sinus Rhythm With ST-T Abnormalities in Leads I, aVL, and V_2_ to V_6_, and ST-Segment Elevation (0.1-0.2 mV) in Lead aVR aVR = augmented voltage right arm; aVL = augmented voltage left arm; CK-MB = creatine kinase-myocardial band; hs-cT = high-sensitivity cardiac troponin.

**Figure 2:**
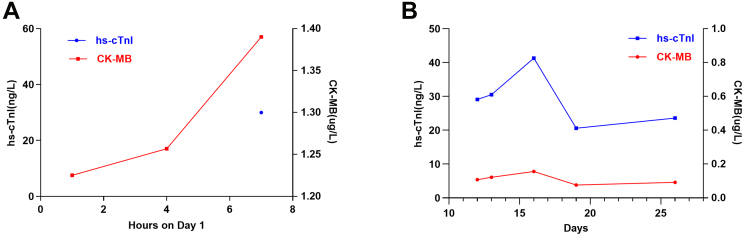
Serial Cardiac Biomarker Changes (A) Upon initial admission, CK-MB levels demonstrated a rapid rise within 7 hours. (B) On day 16, following readmission, a recurrent elevation in both CK-MB and high-sensitivity troponin I (hs-TnI) was observed, coinciding with an episode of chest tightness. CK-MB = creatine kinase-myocardial band; LAD = left anterior descending artery; LCX = left circumflex; RCA = right coronary artery.

A 95-year-old male was admitted following an episode of acute dizziness and nausea, accompanied by hypotension (70/50 mm Hg). The initial electrocardiogram showed atrial fibrillation with widespread ST-segment depression in leads I, augmented left lead, and V_2_-V_6_ and elevation in augmented left lead (Figure 1). Cardiac biomarkers were markedly elevated (troponin I: 5.2 ng/mL [ref. <0.04 ng/mL]; creatine kinase-myocardial band: 57 U/L [ref. <25 U/L]) and demonstrated a dynamic evolving pattern (Figure 2).

## Past Medical History

The patient had a history of transient ischemic attack. There was no history of hypertension or family history of cardiomyopathy.

## Differential Diagnosis

The differential diagnosis consisted of acute coronary syndrome (ACS), atrial fibrillation complicated by rapid ventricular rate, and pulmonary embolism (excluded by imaging).

## Investigations

**Figure 3:**
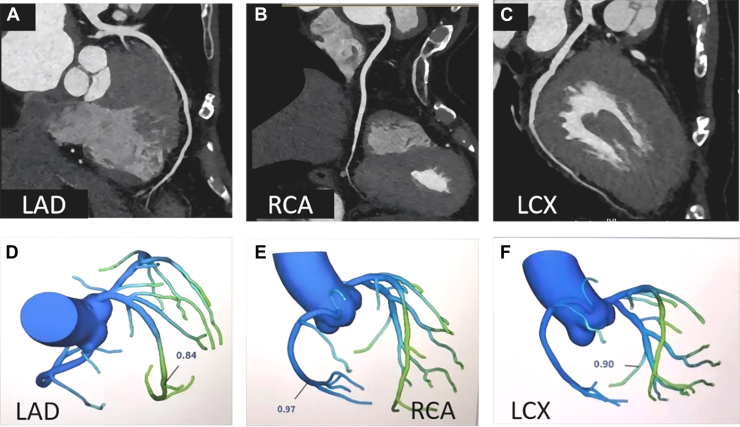
Coronary Computed Tomography Angiography and CT-Derived Fractional Flow Reserve Findings (A to C) Coronary computed tomography angiography images: (A) A myocardial bridge in the mid segment of the left anterior descending artery (LAD). (B and C) No significant stenosis in the left circumflex (LCX) or right coronary artery (RCA). (D to F) CT-derived fractional flow reserve analysis: The values were 0.84 in the LAD (D), 0.90 in the LCX (E), and 0.97 in the RCA (F), indicating no functionally significant ischemia.

During the initial admission, coronary computed tomography angiography revealed no obstructive epicardial disease but identified a myocardial bridge in the mid-left anterior descending artery (Figures 3A to 3C). CT-derived fractional flow reserve values were nonischemic in all major territories (left anterior descending artery: 0.84; left circumflex: 0.90; right coronary artery: 0.97) (Figures 3D to 3F). Computed tomography pulmonary angiography ruled out pulmonary embolism.

Upon readmission, transthoracic echocardiography was performed again to investigate the new systolic murmur and persistent symptoms. This demonstrated:

Asymmetric septal hypertrophy with a maximal wall thickness of 18.7 mm in the basal septum.

Systolic anterior motion of the mitral valve with associated severe mitral regurgitation.

**Figure 4:**
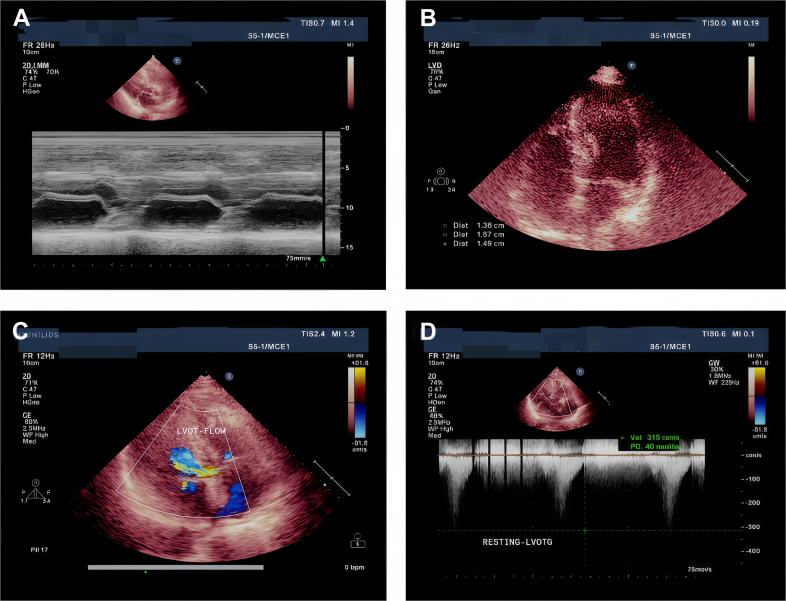
Comprehensive Echocardiogram Confirming Hypertrophic Obstructive Cardiomyopathy Integrated findings include systolic anterior motion of the mitral valve (A), asymmetric septal hypertrophy up to 18.7 mm (B), accelerated LVOT flow on color Doppler (C), and a continuous-wave Doppler-derived peak LVOT gradient of 40 mm Hg at rest (D). LVOT = left ventricular outflow tract.

A moderate left ventricular outflow tract (LVOT) obstruction at rest, with a peak gradient of 40 mm Hg (Figure 4).

These findings enabled the definitive diagnosis of hypertrophic obstructive cardiomyopathy (HOCM).

Subsequent genetic testing using a comprehensive cardiomyopathy gene panel identified a variant of uncertain significance in the *RAF1* gene; no pathogenic variants were found in core hypertrophic cardiomyopathy (HCM) genes (eg, MYH7, MYBPC3).

## Management

Following the initial admission, the patient was discharged on dual antiplatelet therapy (aspirin and clopidogrel) for suspected ACS.

Upon confirmation of HOCM during the second admission, antiplatelet therapy was discontinued due to the absence of significant coronary artery disease. Medical therapy for obstructive HOCM was initiated, starting with bisoprolol 2.5 mg daily. As the patient's symptoms of dizziness and chest tightness persisted, which were attributable to significant LVOT obstruction, the novel cardiac myosin inhibitor mavacamten (5 mg daily) was added to the medication therapy.

## Outcome and Follow-Up

Following the initiation of therapy with bisoprolol and mavacamten, the patient's symptoms of chest tightness and dizziness resolved significantly. He tolerated the medication regimen well without any adverse effects and was discharged in a stable condition on day 31.

The results of genetic testing, which identified a variant of uncertain significance in the *RAF1* gene, were communicated to the patient and his family, with recommendations for familial screening.

A detailed timeline of the patient's clinical course is presented as Timeline of the Case.

## Discussion

HCM is now recognized as one of the most common inherited cardiomyopathies.^1^ Patients typically present with exertional dyspnea secondary to left ventricular outflow tract obstruction, which elevates left ventricular end-diastolic pressure and promotes pulmonary congestion.^2^ Anginal symptoms, often exertion-exacerbated but nitroglycerin-unresponsive, result from myocardial oxygen supply-demand mismatch due to microvascular dysfunction and coronary compression.^3^ This pathophysiology may induce myocardial injury and cardiac biomarker elevation, accompanied by other manifestations including palpitations, fatigue, and presyncope. This report describes an exceptionally late presentation of HOCM in a 95-year-old male, initially misdiagnosed as ACS. The initial clinical picture—ischemic electrocardiography changes, dynamic biomarker elevation, and hypotension—was highly compelling for ACS. The absence of obstructive disease on coronary computed tomography angiography/CT-derived fractional flow reserve provided the first critical clue against significant epicardial coronary disease.

The initial management had limitations, specifically the delay in performing echocardiography and the lack of anticoagulation for atrial fibrillation. This underscores a vital learning point: in any patient presenting with ST-segment elevation myocardial infarction–like features. The initial focus on ruling out coronary disease, while common, led to a delay in diagnosing the underlying cardiomyopathy. The atrial fibrillation was paroxysmal and self-terminating. However, stroke risk should be formally assessed for long-term management.

This case also highlights a potential pitfall in the conventional diagnostic cascade due to suspected ACS. While current guidelines appropriately prioritize the rapid exclusion of obstructive epicardial coronary disease, this approach may, at times, inadvertently delay the consideration of alternative diagnoses such as HOCM. Our experience underscores that cardiac auscultation should be an indispensable component of the initial evaluation, even in emergency settings. The presence of a systolic murmur in this context should serve as a powerful prompt to expedite echocardiography, which can provide a definitive diagnosis and prevent the initiation of potentially inappropriate therapies, such as dual antiplatelet therapy in a patient without significant coronary disease.

The pathophysiology of myocardial ischemia in HOCM is multifactorial, involving microvascular dysfunction and supply-demand mismatch, exacerbated by tachycardia and hypotension, which likely explains this patient's biomarker elevations.^4^ The initiation of mavacamten in this elderly patient was guided by the 2024 American Heart Association/American College of Cardiology guideline,^1^ which recommends adding a myosin inhibitor for patients with obstructive HCM who have persistent symptoms despite the initial medical therapy. Given his significant symptoms and LVOT gradient, the benefit of targeted therapy was deemed to outweigh potential risks. Beyond conventional β-blocker therapy, prompt initiation of mavacamten—a novel cardiac myosin inhibitor—is recommended upon HOCM diagnosis.^4^ This agent targets the underlying pathophysiology by reducing excessive actin-myosin cross-bridge formation, thereby mitigating hypercontractility and improving diastolic function.^5^ The phase III EXPLORER-CN trial (N = 81 symptomatic HOCM patients from 12 Chinese centers) demonstrated significant efficacy and safety of mavacamten vs placebo at 78-week follow-up.^6^ Concurrently, the VALOR-HCM trial showed that mavacamten enabled 82% of surgical candidates to avoid septal reduction therapy.^7^ Mavacamten has been incorporated into international guidelines (Class I recommendation)^1^ and is emerging as a cornerstone of pharmacotherapy for obstructive HCM in China.

## Conclusions

This case demonstrates that HOCM can present in extreme old age, masquerading as ACS. A high index of suspicion, triggered by a systolic murmur and discordant imaging findings, is key to the correct diagnosis. Echocardiography remains the major diagnostic standard. Tailored medical therapy, including novel agents like mavacamten, can be effective and should be considered even in very elderly patients after careful risk-benefit assessment.

## Funding Support and Author Disclosures

This work was supported by the National Key Research and Development Program of China (2022YFC3602400). The authors have reported that they have no relationships relevant to the contents of this paper to disclose.

**Take-Home Messages**

• Hypertrophic obstructive cardiomyopathy is a differential diagnosis for acute coronary syndrome in elderly patients.
• Cardiac auscultation is crucial in differential diagnosis, combined with multimodal cardiac imaging.

**Equipment List:** 

Category	Device/Agent/Test	Specification/Manufacturer/Details
Cardiac imaging & diagnostics	Coronary Computed Tomography Angiography (CCTA) Scanner	Siemens Somatom Force
	CT-derived Fractional Flow Reserve (CT-FFR) Analysis Platform	HeartFlow FFR ∼ CT∼
	Transthoracic Echocardiography Machine	Philips
	12-lead Electrocardiogram (ECG) Machine	Standard hospital system
Pharmacotherapy	Mavacamten	Bristol Myers Squibb
	Bisoprolol	Merck KGaA
	Aspirin	Bayer AG
	Clopidogrel	Sanofi
Genetic testing	Comprehensive Cardiomyopathy Gene Panel	Kingmed diagnostics

This table summarizes the key equipment, pharmacological agents, and diagnostic tests utilized in the management of this case. Specific manufacturers and models are provided where known and relevant to facilitate reproducibility.

**Visual Summary:** Timeline of the Case

Timeline	Events
Day 1	95-year-old male felt acute dizziness and nausea without apparent provocation; blood pressure measured 70/50 mm Hg at that time in emergency. ECG showed: AF and ST-depression (I/aVL/V2-V6), aVR ST-elevation. Biomarkers: troponin I 5.2 ng/mL, CK-MB 57 U/L.
Day 2	CCTA revealed: myocardial bridge (mid-LAD, systolic compression) and CT-FFR showed: LAD 0.84 (excluded ischemia). Discharged with persistent symptoms.
Day 10	Get readmission with angina. Physical examination: Systolic murmur best heard at the left sternal border in the third-fourth intercostal spaces. ECG showed: sinus rhythm, T-inversions (V3-V6).
Day 16	Felt chest tightness. Biomarkers test: high-sensitivity troponin I (hs-TnI) elevated 41.3 ng/L, further reinforcing AMI suspicion.
Day 18	Transthoracic echocardiography definitively established the diagnosis of hypertrophic obstructive cardiomyopathy (HOCM), with a peak pressure gradient of 40 mm Hg.
Day 20	Precision therapy initiation: Bisoprolol 2.5 mg OD and mavacamten 5 mg OD (novel myosin inhibitor)
Day 31	Symptom alleviation and discharged.
Day 47	Genetics test: a VUS in *RAF1* gene (*c.451T>G, p.Phe151Val*).

AF = atrial fibrillation; AMI = acute myocardial infarction; aVL = augmented voltage left arm; aVR = augmented voltage right arm; CCTA = coronary computed tomography angiography; CK-MB = creatine kinase-myocardial band; ECG = electrocardiography; LAD = left anterior descending artery; VUS = variant of uncertain significance.

## References

[1] Ommen S.R., Ho C.Y., Asif I.M., Writing Committee Members (2024). 2024 AHA/ACC/AMSSM/HRS/PACES/SCMR Guideline for the management of hypertrophic cardiomyopathy: a report of the American Heart Association/American College of Cardiology Joint Committee on Clinical Practice Guidelines. J Am Coll Cardiol.

[2] Maron B.J., Desai M.Y., Nishimura R.A. (2022). Diagnosis and evaluation of hypertrophic cardiomyopathy: *JACC* state-of-the-art review. J Am Coll Cardiol.

[3] Shridhar P., Pal S., Clavere N.G., Becker J.R. (2024). Response by Shridhar et al to letter regarding article, "MDM2 regulation of HIF signaling causes microvascular dysfunction in hypertrophic cardiomyopathy". Circulation.

[4] Ho C.Y., Mealiffe M.E., Bach R.G. (2020). Evaluation of mavacamten in symptomatic patients with nonobstructive hypertrophic cardiomyopathy. J Am Coll Cardiol.

[5] Braunwald E., Saberi S., Abraham T.P., Elliott P.M., Olivotto I. (2023). Mavacamten: a first-in-class myosin inhibitor for obstructive hypertrophic cardiomyopathy. Eur Heart J.

[6] Tian Z., Li L., Li X. (2023). Effect of mavacamten on Chinese patients with symptomatic obstructive hypertrophic cardiomyopathy: the EXPLORER-CN randomized clinical trial. JAMA Cardiol.

[7] Desai M.Y., Wolski K., Owens A. (2021). Study design and rationale of VALOR-HCM: evaluation of mavacamten in adults with symptomatic obstructive hypertrophic cardiomyopathy who are eligible for septal reduction therapy. Am Heart J.

